# ZMIZ2 facilitates hepatocellular carcinoma progression via LEF1 mediated activation of Wnt/β-catenin pathway

**DOI:** 10.1186/s40164-024-00475-w

**Published:** 2024-01-22

**Authors:** Yang Ding, Yumei Ning, Hui Kang, Yuan Yuan, Kun Lin, Chun Wang, Yun Yi, Jianghua He, Lurao Li, Xingxing He, Ying Chang

**Affiliations:** 1 Department of Gastroenterology, Zhongnan Hospital of Wuhan University, Wuhan, 430071 China; 2 Hubei Clinical Center and Key Laboratory of Intestinal and Colorectal Diseases, Zhongnan Hospital of Wuhan University, Wuhan, 430071 China

**Keywords:** ZMIZ2, Hepatocellular carcinoma, Prognosis, LEF1, Wnt/β-catenin pathway

## Abstract

**Background:**

Hepatocellular carcinoma (HCC) is one of the most common malignancies with a high lethality rate. ZMIZ2 is a transcriptional co-activator implicated in various human diseases. However, the role and molecular mechanism of ZMIZ2 in HCC remains to be elucidated.

**Methods:**

The expression and prognostic value of ZMIZ2 in HCC was excavated from public databases and explored by bioinformatic analysis. Then the expression of ZMIZ2 and related genes was further validated by quantitative RT-PCR, western blotting, and immunohistochemistry. Loss and gain-of-function experiments were performed in vitro and in vivo to investigate the function of ZMIZ2 in HCC. In addition, transcriptome sequencing and immunoprecipitation was conducted to explore the potential molecular mechanisms of ZMIZ2.

**Results:**

ZMIZ2 was highly expressed in HCC and associated with poor prognosis. Silencing ZMIZ2 significantly inhibited HCC cell proliferation, cell cycle process, migration, and invasion in vitro, and also inhibited the progression of HCC in vivo. Additionally, ZMIZ2 expression was correlated with immune cell infiltration in HCC samples. Somatic mutation analysis showed that ZMIZ2 and TP53 mutations jointly affected the progression of HCC. Mechanistically, ZMIZ2 interacted with LEF1 to regulate malignant progression of HCC by activating the Wnt/β-catenin pathway.

**Conclusion:**

ZMIZ2 was overexpressed in HCC and associated with poor prognosis. The overexpression of ZMIZ2 was corelated with malignant phenotype, and it facilitated HCC progression via LEF1-mediated activation of the Wnt/β-catenin pathway. Furthermore, ZMIZ2 could be served as a prognostic biomarker and a new therapeutic target for HCC.

**Supplementary Information:**

The online version contains supplementary material available at 10.1186/s40164-024-00475-w.

## Introduction

Hepatocellular carcinoma (HCC) is a worldwide common malignancy with high incidence rate and high mortality [1]. Aggressive is widely considered as the main driving force for HCC metastasis and recurrence [2], and the rapid disease progression caused by tumor recurrence is the primary cause of the mortality rate [3]. In addition, the majority of HCC patients have a poor prognosis due to insufficient early diagnosis and limited effective treatment options for advanced HCC [4, 5], which drives global researchers to reveal deeper into the molecular mechanism of hepatocarcinogenesis and explore new treatment strategies.

HCC is characterized by the decreased genomic stability and accumulation of somatic DNA alterations such as TP53 mutations [6]. Besides, sustained activation of oncogene signaling such as RHO GTPase related signaling [7], HGF/c-MET signaling [2], PI3K/AKT signaling [3], NRAS/ERK signaling [8], is a key driver of hepatocellular carcinoma (HCC) progression. Hyperactivation of the Wnt/β-catenin signaling is an early event in HCC pathogenesis [9, 10]. Approximately 50% of HCC exhibit activation of the Wnt/β-catenin signaling pathway due to mutations in CTNNB1, AXIN1 or APC [11]. Agents such as salinomycin and NVP-TNKS656 targeting the upstream molecules of Wnt/β-catenin signaling have been developed. However, these agents may be ineffective when the pathway is activated downstream. Blocking the interactions between β-catenin and its transcriptional partners with agents such as PRI-724, ICG001, and isoquercitrin is a promising therapeutic approach [12]. Therefore, extensive studies and the ongoing clinical trials highlight the promise for developing novel therapeutic strategies targeting the Wnt/β-catenin pathway.

Zinc finger MIZ type containing 2 (ZMIZ2) is a co-activator of transcription and an inhibitor of activated STAT-like proteins (PIAS) [13]. The protein also possesses an intrinsic transcription activation domain (TAD) that enhances the transcriptional activity of nuclear hormone receptors and other transcription factors [14–17]. While aberrant ZMIZ2 expression has related to several cancer types, such as prostate cancer [13], breast cancer [18] and colorectal cancer [19], its potential involvement in HCC remains unclear.

Lymphoid enhancer-binding factor-1 (LEF1) is a member of the LEF1/T-cell factor family and serves as a nuclear effector in the Wnt/β-catenin signaling pathway [20]. It is characterized by a highly conserved high-mobility group DNA-binding domain [20, 21]. As a transcription regulatory factor, LEF1 controls the expression of various genes and influences the progression of multiple malignancies [22–25]. Prior studies have shown that LEF1 promotes cell self-renewal ability, dedifferentiation, invasion, and drug resistance in HCC [22, 26–28]. As a key player in the Wnt/β-catenin pathway, LEF1 binds to accumulated β‐catenin in the nucleus and promotes the transcription of Wnt‐responsive genes, which in turn drives cell growth and malignant progression [29]. However, whether LEF1 interacts with ZMIZ2 to modulate the Wnt/β-catenin pathway is yet to be determined.

The TP53 gene is the most commonly mutated gene in cancer [30, 31]. It is reported that TP53 mutational inactivation causes β-catenin accumulation and activates the Wnt signaling pathway [32–34]. The relationship between the role of ZMIZ2 and TP53 mutations remains unclear. Transcriptional co-activators, such as YAP/TAZ and CBP/p300, have been implicated in the establishment of tumor microenvironment (TME) and immune escape [35–37]. However, the role of ZMIZ2 in HCC tumor immunity deserves further exploration.

In the present study, the expression and clinical significance of ZMIZ2 in HCC were explored by bioinformatics analysis and experimental validation. In particular, loss and gain-of-function study, transcriptome sequencing and co-immunoprecipitation experiments were conducted to explore the significant role of ZMIZ2 in activating the Wnt/β-catenin signaling pathways to promote HCC progression.

## Materials and methods

### Patients specimens and clinical data collection

Eight pairs of primary liver cancer tissues and adjacent non-tumor tissue samples were obtained from Zhongnan Hospital of Wuhan University. Informed consent was obtained from patients participating in this study. Furthermore, detailed clinical information of these HCC patients was obtained from the electronic medical record system of Zhongnan Hospital. This study was also approved by the ethics committee of Zhongnan Hospital (Approval No.2022011K).

### Cell culture

Human immortalized liver cell line of L02 and liver cancer cell lines including Huh7, Hep3B, HCCLM3, HepG2, SK-hep1, MHCC-97 H were were routinely cultured in high glucose DMEM (Gibco, USA) or MEM (Gibco, USA) containing 10% fetal bovine serum (Gibco, USA). All cells were nurtured in a saturated humidity at 37 °C with 5% CO_2_.

### RNA extraction, reverse transcription and quantitative real-time PCR

Total RNA was extracted from cells and tissues using Trizol Reagent (Invitrogen, USA). The Nanodrop 2000 spectrophotometer (ThermoFisher, USA) was used to measure the quantity and quality of RNA. cDNA was produced through a reverse transcription process with the TOYOBO ReverTra Ace Kit (Toyobo, Japan). Gene expression was accomplished using UltraSYBR Mixture (CWbio, China) and the CFX96 Touch Real-Time PCR system (Biorad, USA). The mRNA expression levels of ZMIZ2 and related genes were standardized to GAPDH. The relative quantification was calculated using the 2^−△△CT^ method. Primers designed for RT-PCR in this research are listed in Additional file 2: Table S2.

### EDU assay

All transfected with siRNA, plasmid cells were seeded into 96-well plates at 4 × 10^3^ cells/well and incubated for 24 h. The proliferation ability was detected with an EDU assay kit (#C10310-1, RIBO, Guangzhou, China) following the manufacturer’s instructions. Images were acquired using a fluorescence microscope (Olympus, Shanghai, China), and the percentage of EDU positive cells was calculated using the formula EDU-positive cell count/total cell count × 100%.

### Cell counting kit 8 assay

Transfected correspondingly siRNA, plasmid or control to Huh7, Hep3B, SK-hep1 and HepG2 cells for 24 h, Thereafter, 2000 and 4000 transfected cells were seeded into separate 96-well plates. Cell viability was assessed with the Cell Counting Kit-8 (CCK8) (#CK04, Dojindo, Japan) according to the manufacturer’s instructions. CCK-8 reagent was added at 0 h,24 h,48 h,72 h,96 h. After 2 h of incubation, absorbance reading was tested on a microtiter plate reader at 450 nm (ELX-800, BioTek, USA). Then the growth curves were plotted according to the OD values.

### Luminescent cell viability assay

Cell transfection, counting, and seeding are the same as CCK8. The CellTiter-Glo^®^ Luminescent Cell Viability Assay Kit (#G7570, Promega, USA) was used to determine the intracellular ATP and reflect cell viability. First, 100 µl CellTiter-Glo solution was directly mixed with the culture medium and incubated at room temperature for 10 min. Following this, the intracellular ATP luminescence was recorded using Victor Nivo^®^ multimode plate reader (Perkin-Elmer, Midrand, South Africa). Add reaction solution at 0 h, 24 h, 48 h, 72 h, and 96 h to detect the luminescence signal.

### Wound healing, migration and matrigel invasion assays

The 200 µl pipette tip was employed to create wounds when 90% of the tumor cells had merged following transfection. The previous cell culture medium was replaced by a medium containing 2% fetal bovine serum. At 0 h, 24 h, and 48 h, the wounds were noted by a microscope and photographed, respectively. For migration assays, 5 × 10^4^ cells suspended in serum-free medium and 400 µl of cell suspension were seeded into the upper chamber. Then the lower chamber was filled with 600 µl medium containing 10% FBS, which acted as a chemoattractant. After 24 to 48 h, the cells were wiped off the upper chamber (6.5 mm. in diameter, 8.0 μm pore size, Corning, USA) with a cotton swab. The invaded cells to the underside of the membrane were fixed with 4% paraformaldehyde and stained with 0.1% crystal violet (#C0121, Beyotime, Shanghai, China), then counted in three randomly selected microscopic fields (200×) per filter. For Matrigel invasion assays, we paved Matrigel (#356234, Corning, USA) in the surface of upper chamber and 1 × 10^5^ cells were seeded. The remaining steps were same as migration assays.

### Flow cytometry analysis

Huh7, Hep3B, SK-hep1 and HepG2 cells were transfected with required siRNA, plasmid, or control, then cell apoptosis assay was performed with the Annexin V-FITC/PI Apoptosis Detection Kit (#4101-2, BestBio, China). The cells were stained with PI to conduct cell cycle analysis using cell cycle staining kit (#CCS012, Multisciences, China) according to the manufacturer’s protocol. The cells were analyzed by flow cytometry (Cytoflex Beckman, China).

### Immunohistochemistry (IHC)

HCC tissues and mouse tumor tissues were fixed by 4% paraformaldehyde at room temperature for 24 h. Embedded in paraffin, dewaxed and rehydrated, antigen extraction was performed using corresponding antigen retrieval solution at 100 °C for 20 min. Later, 3% hydrogen peroxide solution was applied to inhibit endogenous peroxidase activity for 15 min at room temperature. The slides were incubated with 5% normal goat serum in PBST for 1 h at room temperature to prevent nonspecific binding of antibodies. After that, samples are incubated with the designated primary antibody overnight at 4 °C. Each slide was incubated with the corresponding HRP-labeled secondary antibody, signal generated with DAB solution, and counterstained with hematoxylin after three TBST washings. Image J was used to analyze the intensity of IHC staining. The antibodies involved in this study are specified in Additional file 2: Table S1.

### Animal experiment

Six-week-old male BALB/c nude mice were bought from Charles River Labs (Beijing, PR China). All animal experimental procedures were approved by the Experimental Animal Welfare Ethics Committee, Zhongnan Hospital of Wuhan University (ZN2022088). The six-week-old male mice were randomized into different groups. SK-hep1 cells (5 × 10^6^ cells/mice) with stable knockdown ZMIZ2 and control SK-hep1 cells were subcutaneously injected into the nude mice. After tumor formation, tumor volume (mm^3^) was measured by Vernier calliper every other day and calculated by the formula (length × width × width)/2. All the mice were sacrificed after 37 days. The tumor samples were then fixed in 4% paraformaldehyde and embedded in paraffin for IHC analysis.

### Coimmunoprecipitation (Co-IP)

Protein samples were first prepared, then LEF1 antibody (1:50, ABclonal, A0909) and IgG were added separately, and the tubes were gently vortexed and incubated overnight at 4 °C. The next day, 5 µl ProteinA/ProteinG (CST, 70024) Magnetic Beads were added to each tube and mixed gently at 4 °C for 3 h. Then the precipitate was centrifuged at 4 °C and washed with 1 × wash buffer. Next, the proteins were eluted from the beads using 1× SDS loading buffer and boiled at 95 °C for 10 min. Finally, western blot assays were performed for further verification.

### Western blot

Cells and HCC samples were collected using RIPA buffer (P0013B, Beyotime, China) containing protease inhibitor (#HY-K0010, MCE, China) and phosphatase inhibitor (#HY-K0021&#HY-K0022, MCE, China). Proteins were centrifuged at 15,000 rpm for 20 min at 4 °C; Then supernatants were collected, and protein concentrations were calculated by the BCA Kit (P0012, Beyotime, China). RIPA buffer was mixed with protein loading buffer and protein was denatured at 100 °C for 5 min. An equal amount of proteins was separated using SDS-polyacrylamide gels and then transferred to polyvinylidene fluoride (PVDF) membranes (Millipore, USA). Membranes were blocked for 2 h with 5% skimmed milk and incubated with primary antibodies overnight at 4 °C. The membrane was incubated with a secondary antibody at room temperature for 1.5 h After three washes with TBST. Protein bands were obtained using an enhanced chemiluminescence (ECL) kit (SQ202, Epizyme, China) using GENESys (Synoptics Ltd, China). The antibodies involved in this study are prescribed in Additional file 2: Table S1.

### Dataset analysis

Gene expression of ZMIZ2 of HCC and normal tissues were obtained from publicly available datasets, including The Cancer Genome Atlas (TCGA)-LIHC cohort and Gene Expression Omnibus (GEO) datasets (accession number: GSE54236, GSE55092 and GSE144269). The gene expression profiles and corresponding clinical information were downloaded from the UCSC Xena browser (https://xenabrowser.net) and GEO database (http://wwwncbinlmnih.gov/geo/), respectively.

### Tumor somatic mutation and immune infiltration analysis

Somatic mutation data of the TCGA-LIHC cohort were downloaded from TCGA database (http://cancergenome.nih.gov/). The top 15 mutation genes were obtained and then compared between ZNIZ2 subgroups. According to the definition of the tumor mutation burden (TMB) in published literature [16], we evaluated the TMB score of patients in the TCGA-LIHC cohort. The relative abundance of 28 immune cell infiltration in each HCC sample was quantified via ssGSEA algorithm by previously described methods [38].

### Functional annotation, gene set variation analysis (GSVA), and gene set enrichment analysis (GSEA)

To elucidate the role of ZMIZ2 in HCC, gene ontology (GO) functional annotation analysis was performed based on the top 200 differentially expressed genes (DEGs) (high-ZMIZ2 versus low-ZMIZ2). The DEGs were screened using “limma” R package. GSVA and GSEA was also conducted between ZMIZ2 subgroups via the “clusterProfler” and “GSVA” R packages, respectively. The gene set of “H.all.v7.4” was downloaded from the Molecular Signatures Database [39].

### Statistical analysis

Data were represented as means ± standard deviation. Data analyses, including normality test, paired t-test, Spearman correlation test, Kaplan-Meier method, and univariate and multivariate analyses by Cox proportional hazard regression models, were performed using R (version 4.1.1), SPSS version 20.0 (IBM SPSS, Armonk, NY, USA) and GraphPad PRISM version 9.0 (GraphPad Software Inc., San Diego, CA, USA) software. Differences between data were considered statistically significant at *P* < 0.05.

## Results

### ZMIZ2 was overexpressed in HCC and high ZMIZ2 was correlated with poor clinical prognosis

**Fig. 1:**
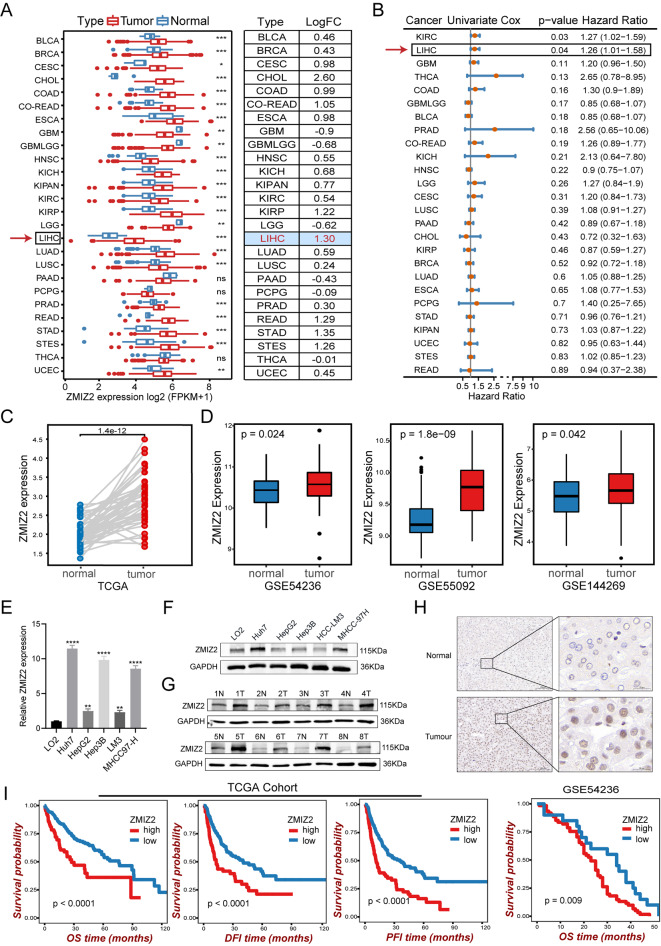
ZMIZ2 is overexpressed in HCC and high ZMIZ2 is correlated with poor clinical prognoses. (**A**) ZMIZ2 mRNA expression levels in 26 common human tumors were analyzed based on data from The Cancer Genome Atlas (TCGA) database. The log (fold change) of each type cancer versus adjacent normal tissue was shown in the right table. (**B**) The risk ratio of ZMIZ2 in 26 common human tumors was analyzed via a forest plot. (**C**) Paired Student’s t-test analysis of ZMIZ2 expression in paired samples of TCGA-LIHC cohort. (**D**) ZMIZ2 mRNA expression levels in HCC were significantly higher than that in normal tissues according to the GSE54236, GSE55092, and GSE144269 datasets. (**E, F**) mRNA expressions (**E**) and protein levels (**F**) of ZMIZ2 were decreased in a panel of HCC cell lines compared with that in immortalized LO2 human hepatocytes. (**G, H**) The protein expression of ZMIZ2 in HCC tissues and adjacent normal tissues was detected by western blotting (**G**) and immunohistochemistry (**H**) analysis. Scale bars: 100 or 20 μm. (**I**) Kaplan–Meier plots representing the prognostic values of ZMIZ2 in HCC patient from the TCGA and GSE54236 cohorts. Statistical analysis was conducted using the log-rank test. **p < 0.01; ***p < 0.001; **** *p* < 0.0001

**Fig. 2:**
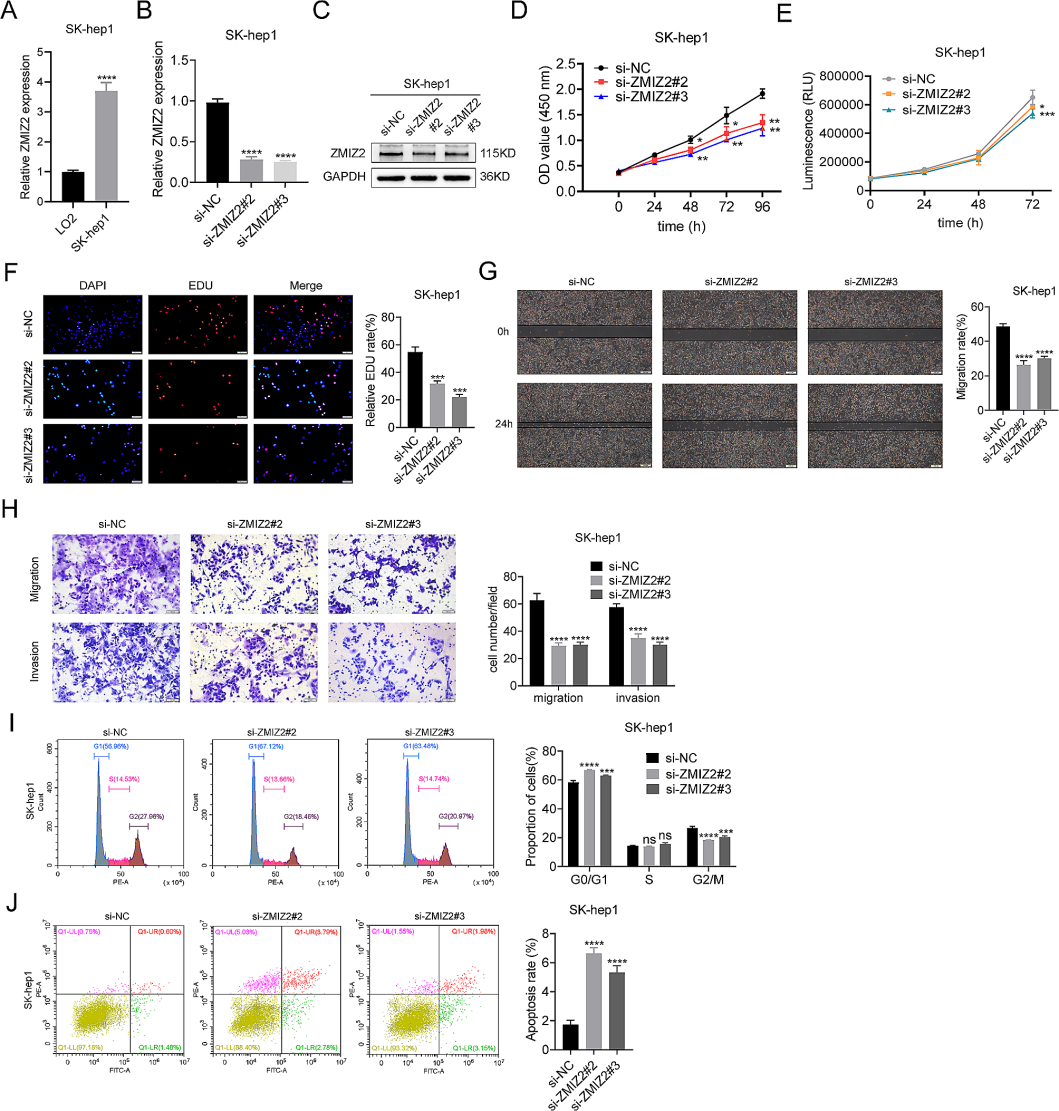
ZMIZ2 promotes the malignant biological behavior of SK-hep1 cells. (**A**) The mRNA expressions of ZMIZ2 were highly increased in SK-hep1 cell compared with immortalized LO2 human hepatocytes. (**B**) The efficiencies of ZMIZ2 silencing in SK-hep1 cell were validated by qRT-PCR. (**C**) The efficiencies of ZMIZ2 silencing in SK-hep1 cell were validated by Western blotting. (**D-F**) CCK8 assay, Luminescent Cell Viability Assay and EDU assays were performed to identify proliferation after ZMIZ2 knockdown in SK-hep1 cells. (**G-H**) Wound healing, migration, and invasion assays were performed to identify metastasis ability after ZMIZ2 knockdown in SK-hep1 cells. (**I**) Flow cytometry results showing the cell cycle distribution after ZMIZ2 knockdown in SK-hep1 cells. (**J**) Flow cytometry results showing the percentage of apoptotic cells after ZMIZ2 knockdown in SK-hep1 cells. *p < 0.05; **p < 0.01; ***p < 0.001; **** *p* < 0.0001; ns, not significant

A pan-cancer analysis of ZMIZ2 mRNA expression levels in 26 common human cancers showed that ZMIZ2 was overexpressed in various tumors compared with adjacent normal tissue (logFC > 0). Of note, ZMIZ2 mRNA expression levels were remarkably higher in HCC (*n* = 369) than that in normal tissues (*n* = 50) (logFC = 1.30, *P* < 0.001) (Fig. 1A). Univariate Cox analysis revealed that high ZMIZ2 expression was associated with poor prognosis in Liver hepatocellular carcinoma (LIHC) (HR = 1.26, *p* = 0.04) (Fig. 1B). Paired Student’s t-test analysis confirmed the ZMIZ2 mRNA expression pattern in TCGA-LIHC (Fig. 1C). In addition, analysis in three independent GEO HCC cohorts (GSE54236, GSE55092, and GSE144269) further confirmed that ZMIZ2 was significantly upregulated in HCC tissues (Fig. 1D). Furthermore, the mRNA and protein expression of ZMIZ2 was highly increased in several hepatoma cell lines compared with immortalized LO2 human hepatocytes (Figs. 1E and F and 2A). Higher protein levels were also found in eight HCC specimens than in adjacent normal tissues via western blot (Fig. 1G) and IHC (Fig. 1H). Regarding the potential indicating role of ZMIZ2 in clinical prognosis, Kaplan-Meier survival analysis showed that higher ZMIZ2 expression was associated with shorter overall survival times (Fig. 1I), and subsequent univariate Cox regression analysis indicated that ZMIZ2 expression was a risk factor for HCC prognosis (Table 1). The correlation between ZMIZ2 and clinical features of HCC patients in TCGA-LIHC was presented in Table 2. Overall, these results indicated that ZMIZ2 was upregulated in HCC and elevated expression of ZMIZ2 was associated with worse clinical outcomes.

**Table 1:** Correlation of clinicopathologic characteristics and ZMIZ2 in TCGA-LIHC cohort

Characteristics	N	Percent (%)	ZMIZ2 level		*P* Chi squared test
			Low	High	
**Total cases**	369	100	184	185	
**Gender**					0.683
Male	249	67.48	126	123	
Female	120	32.52	58	62	
**Age**					0.754
< 60	169	45.80	86	98	
≥ 60	199	53.93	98	101	
**Clinical stage**					**0.056**
I	171	46.34	96	75	
II	86	23.31	38	48	
III	83	22.49	35	48	
IV	5	1.36	4	1	
**Pathology T stage**					**0.060**
T1	181	49.05	101	80	
T2	94	25.47	40	54	
T3	78	21.14	35	43	
T4	13	3.52	5	8	
Tx			3	0	
**Pathology N stage**					1
N0	250	67.75	125	125	
N1	4	1.08	2	2	
**Pathology grade**					0.020*
G1-2	232	62.87	126	106	
G3-4	132	35.77	55	77	
**Vascular invasion**					0.620
None	206	55.83	114	92	
Micro	93	25.20	46	47	
Macro	14	3.79	7	7	

* *p*-value < 0.05

**Table 2:** Univariate and multivariate analyses of various clinical features with LIHC Cox-regression analysis

Characteristics	Univariate analysis			Multivariate analysis		
	*P*	Hazard ratio	95%CI	*P*	Hazard ratio	95%CI
ZMIZ2 (high)	**0.021**	1.408	1.05–1.88	0.522	1.127	0.78–1.63
Gender (female)	0.292	0.826	0.58–1.18			
Age (≥ 60)	0.278	1.214	0.86–1.72			
Stage (III-IV)	**< 0.001**	2.486	1.71–3.61	0.225	3.413	0.47–24.92
Grade (G3-4)	0.539	1.121	0.78–1.61			
T stage (T3-4)	**< 0.001**	2.588	1.82–3.69	0.623	0.603	0.08–4.52
Vascular invasion	**0.024**	2.489	1.13–5.49	0.106	1.954	0.87–4.42

**Bold** indicates statistical differences (*p* < 0.05)

### ZMIZ2 promotes HCC cell proliferation

**Fig. 3:**
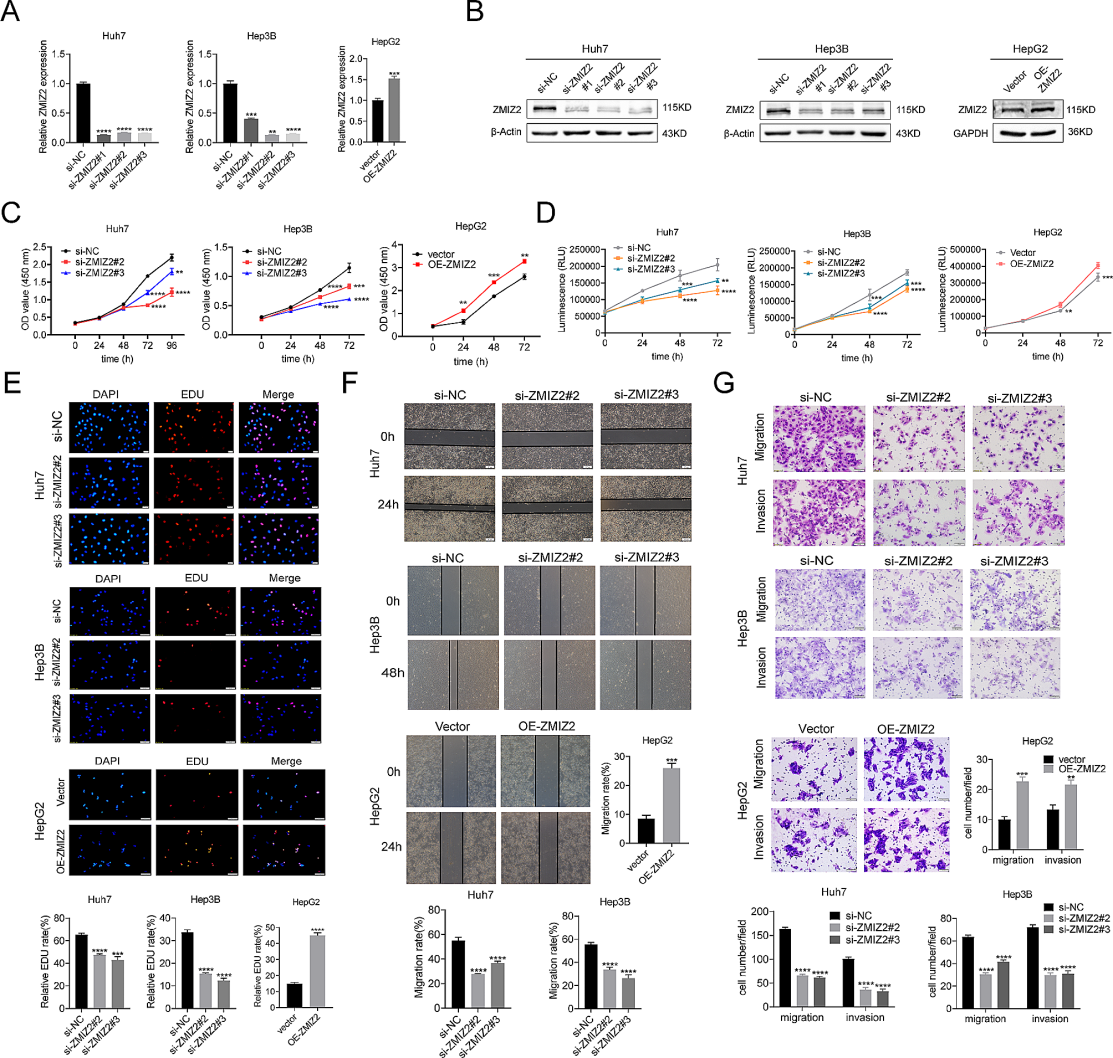
ZMIZ2 promotes HCC cell proliferation, migration, and invasion. (**A-B**) The efficiencies of ZMIZ2 silencing and ZMIZ2 overexpression were validated by qRT-PCR and Western blotting. (**C-E**) CCK8 assay, Luminescent Cell Viability Assay and EDU assays were performed to identify proliferation after ZMIZ2 knockdown in Huh7 and Hep3B cell, ZMIZ2 overexpression in HepG2 cell. (**F-G**) Wound healing, migration, and invasion assays were performed to identify metastasis ability after ZMIZ2 knockdown in Huh7 and Hep3B cell and ZMIZ2 overexpression in HepG2 cell. **p < 0.01; ***p < 0.001; **** *p* < 0.0001

To investigate the role of ZMIZ2 in the development of HCC, we chose several HCC cells lines to perform related experiments. Among the six HCC cell lines, Huh7, Hep3B, MHCC97H and SK-hep1 cells showed the highest ZMIZ2 expression at mRNA levels, while HepG2 and LM3 cells showed relatively low expression levels of ZMIZ2 (Figs. 1E and 2A). Therefore, Huh7, Hep3B and SK-hep1 were selected for ZMIZ2 silencing, and HepG2 was chosen for ZMIZ2 overexpression. Three siRNAs (si-ZMIZ2#1, si-ZMIZ2#2, si-ZMIZ2#3) and plasmids (OE-ZMIZ2) were transfected into the cells. The efficiencies of ZMIZ2 silencing and overexpression were validated by qRT-PCR and Western blotting (Figs. 3A and B and 2B and C). The results showed that si-ZMIZ2#2 and si-ZMIZ2#3 had a better inhibition efficiency (Fig. 3A, B), which were chosen for subsequent experiments. CCK8 assay, Luminescent Cell Viability Assay and EDU assay revealed that ZMIZ2 knockdown significantly impaired HCC cell proliferation (Figs. 3C-E and 2D-F). In contrast, ZMIZ2 overexpression promoted the HCC cell proliferation (Fig. 3C-E).

### ZMIZ2 promotes HCC cell migration and invasion in vitro

Previous studies have shown that ZMIZ2 plays a role in the invasion and migration of breast cancer cells [18]. Herein, the wound healing assays also revealed a significant reduction of migration in the siZMIZ2 group compared with the control group (Figs. 3F and 2G). The transwell assay confirmed that downregulation of ZMIZ2 suppressed the migration and invasion ability of Huh7, Hep3B and SK-hep1 cells (Figs. 3G and 2H). Conversely, overexpression of ZMIZ2 promoted the migration and invasion ability of HepG2 cells (Fig. 3F, G).

### ZMIZ2 downregulation arrested cell cycle and promoted apoptosis of HCC cells

**Fig. 4:**
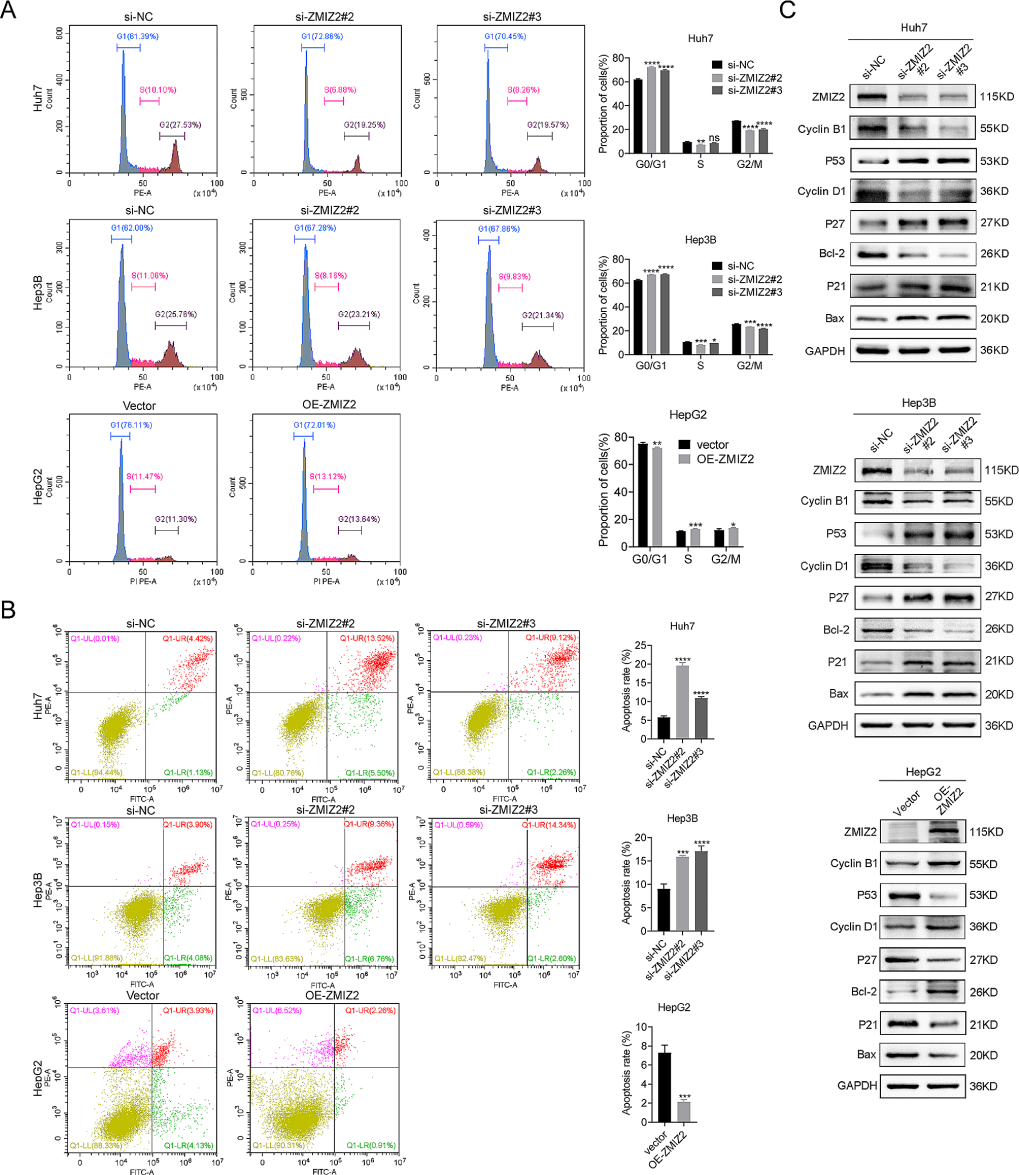
ZMIZ2 downregulation arrested cell cycle and promoted apoptosis of hepatoma cells. (**A**) Flow cytometry results showing the cell cycle distribution after ZMIZ2 knockdown in Huh7 and Hep3B cell, ZMIZ2 overexpression in HepG2 cell. (**B**) Flow cytometry results showing the percentage of apoptotic cells after ZMIZ2 knockdown in Huh7 and Hep3B cell, ZMIZ2 overexpression in HepG2 cell. (**C**) The expression of genes related to the cell cycle and apoptosis was detected by Western blotting. *p < 0.05; **p < 0.01; ***p < 0.001

To determine the potential biological functions of ZMIZ2 in HCC, we performed GSEA and found that patients with high ZMIZ2 expression were significantly enriched in the cell cycle signaling and apoptosis pathways in the TCGA cohort (Additional file 1: Fig. S1). Subsequently, the flow cytometry analysis revealed the suppression of ZMIZ2 could significantly block the process of cell cycle and particularly induced cell cycle G0/G1 phase arrest in Huh7, Hep3B and SK-hep1 cells (Figs. 4A and 2I). Besides, the percentage of apoptosis cells was significantly increased by ZMIZ2 silencing (Figs. 4B and 2J). In contrast, ZMIZ2 overexpression showed opposite results (Fig. 4A, B). Western blot showed that ZMIZ2 silencing reduced the expression of Bcl-2, Cyclin B1, and Cyclin D1 but increased Bax, p21, p27, and p53 protein levels (Fig. 4C), indicating that ZMIZ2 was associated with cell cycle and apoptosis.

### ZMIZ2 enhanced the oncogenesis of HCC cells in vivo

**Fig. 5:**
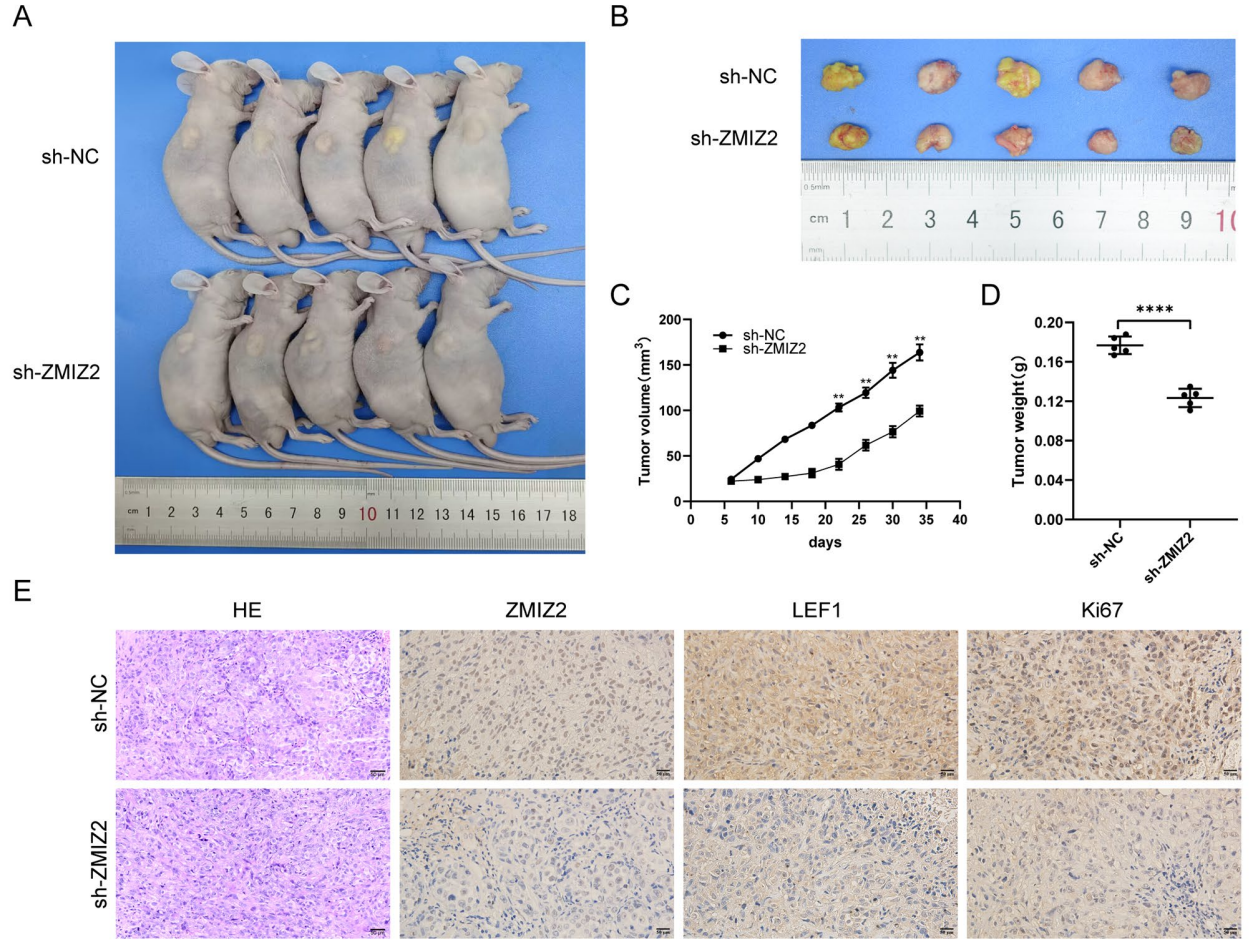
ZMIZ2 enhanced the oncogenesis of HCC cells in vivo. (**A-B**) Representative images of tumors formed in nude mice injected subcutaneously with the SK-hep1 and SK-hep1-shRNA cells (*N* = 5). (**C**) Tumor growth curves in the indicated groups. (**D**) Assessment of the weight of the dissected tumors. (**E**) Representative images of IHC staining of ZMIZ2, LEF1, and Ki67 in xenograft tumors. Scale bars: 50 μm. **p < 0.01; ***p < 0.001; **** *p* < 0.0001

In order to explore the function of ZMIZ2 in vivo, we utilized lentivirus-mediated ZMIZ2 knockdown to establish sh-ZMIZ2 SK-hep1 cells. These cells were injected subcutaneously into the axilla of nude mice. We found the tumor growth rate was significantly slowed in the sh-ZMIZ2 group compared to the control group (Fig. 5A, B). The tumor size and weight were significantly reduced after ZMIZ2-knockdown (Fig. 5C, D), indicating that ZMIZ2 deficiency inhibited HCC tumor development in vivo. IHC staining also revealed that the ZMIZ2 knockdown group had lower levels of ZMIZ2, LEF1, and Ki-67 staining intensity compared to the normal tissues (Fig. 5E). Taken together, these findings suggest that ZMIZ2 plays a significant role in promoting oncogenesis and progression of HCC in vivo.

### Different patterns of tumor somatic mutations in patients between ZMIZ2 expression subgroups

**Fig. 6:**
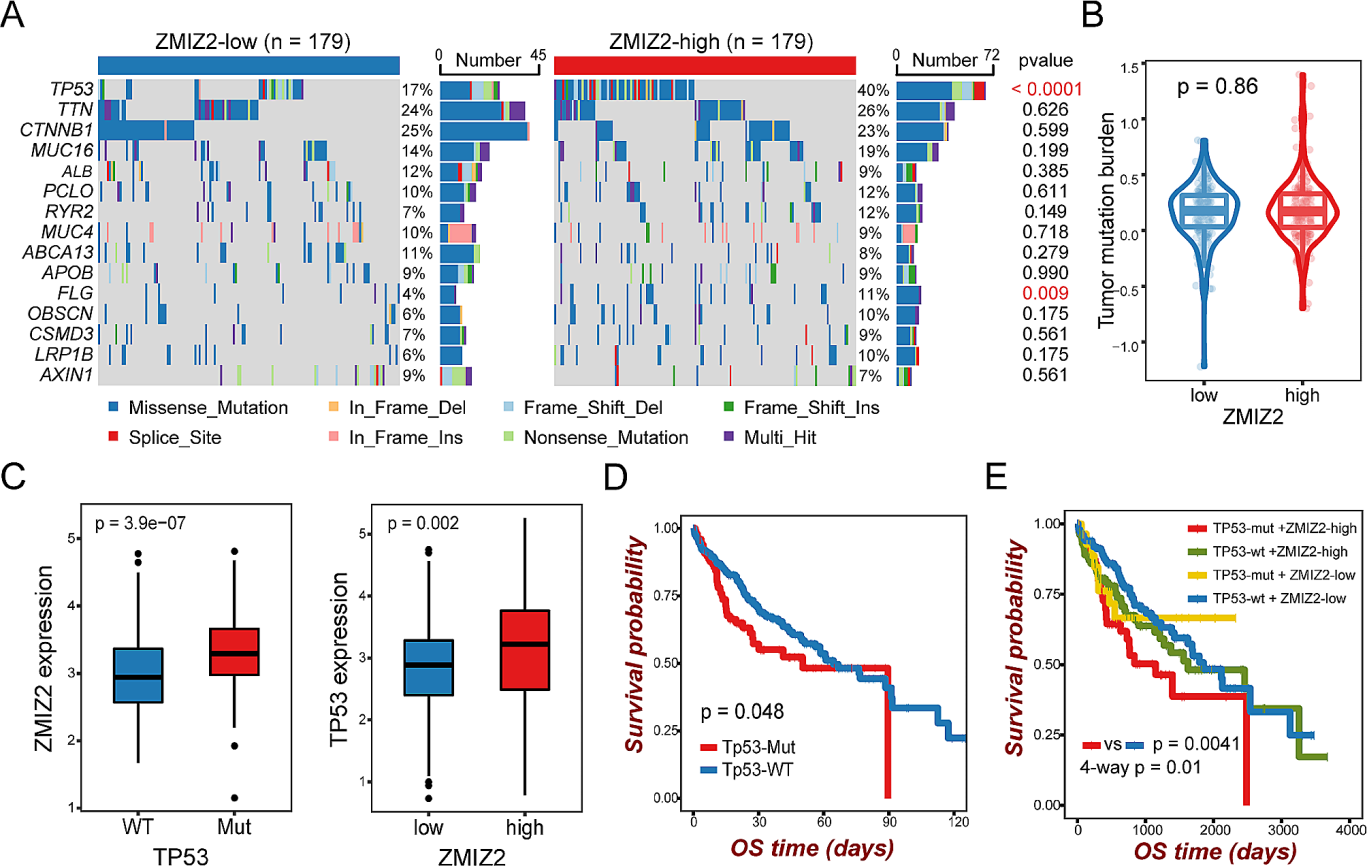
Association between tumor somatic mutations and ZMIZ2 expression. (**A**) The waterfall plots showed top 15 gene alterations in the ZMIZ2 high and low expression group, respectively. (**B**) Violin plot shows no significant difference in tumor mutational burden between ZMIZ2 expression subgroups. (**C**) Box plots showing correlation of ZMIZ2 expression and TP53 mutation. (**D**) Kaplan-Meier overall survival curves of HCC patients with or without TP53 gene mutation. (**E**) Kaplan-Meier overall survival curves of HCC patients divided by TP53 mutation status and ZMIZ2 expression

We next investigated the tumor somatic alterations between high and low ZMIZ2 expression groups. The top 15 variant mutations were identified in 358 HCC patients of the TCGA-LIHC cohort (Fig. 6A). We found no significant difference in tumor mutation burden score between the two subgroups (Fig. 6B). However, TP53 mutations (*p* < 0.0001) were more frequent in samples with high ZMIZ2 expression (Fig. 6A), and ZMIZ2 expression was higher in TP53-mutated samples (Fig. 6C). Patients without TP53 mutation had a better prognosis than those with TP53 mutation (Fig. 6D), which is consistent with previous studies [40, 41]. Notably, patients with TP53 mutation as well as high ZMIZ2 expression had the poorest prognosis among all subgroups (Fig. 6E). These results indicated that ZMIZ2 and TP53 mutations may have a potential cooperative effect on the development and prognosis of HCC.

### Correlation between ZMIZ2 expression and immune cells infiltration in HCC

**Fig. 7:**
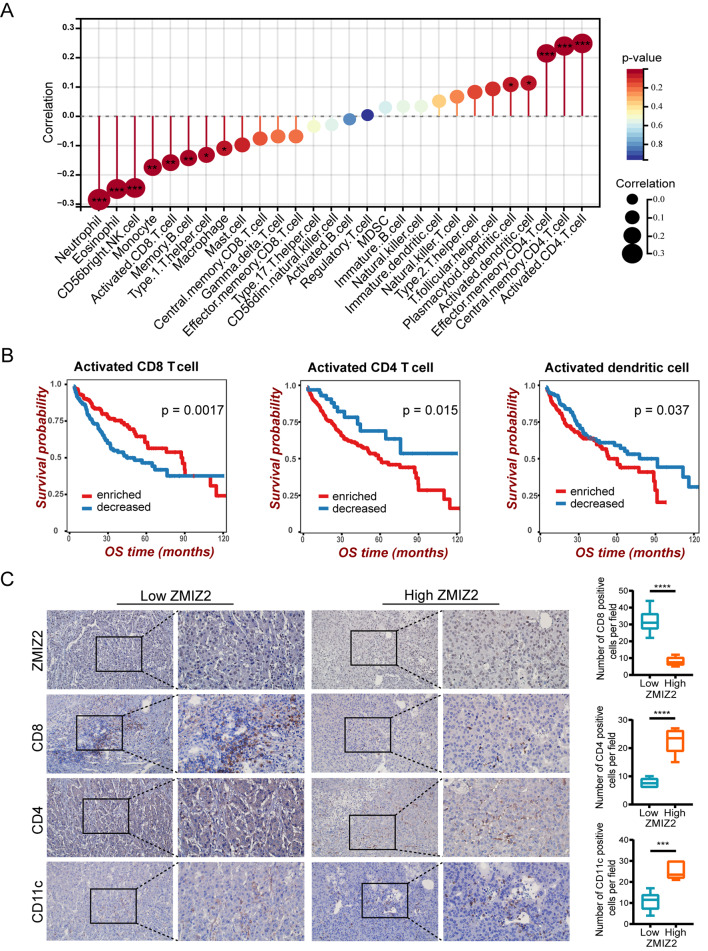
Association of ZMIZ2 expression with immune cell infiltration in HCC. (**A**) Forest plotters shows the correlation between ZMIZ2 expression and 28 types of immune cells according ssGSEA algorithm. (**B**) Kaplan-Meier curves showing the prognostic effects of immune cell infiltrations in HCC. (**C**) Immunohistochemistry staining shows the association of ZMIZ2 expression with immune cell infiltration in HCC clinical samples. Scale bars: 100 or 50 μm. ***p < 0.001; **** *p* < 0.0001

Next, we analyzed the relationship between ZMIZ2 expression and immune cells infiltration by the ssGSEA algorithm. As shown in Fig. 7A, there was a positive correlation between ZMIZ2 expression and the infiltration of CD4 T cells and dendritic cells (DCs). In contrast, ZMIZ2 expression was significantly negatively correlated with the infiltration of multiple anti-tumor immune cells, such as NK cells, CD8 T cells, and Th1 cells. Consistently, the infiltration levels of those anti-tumor immune cells were significantly decreased in the ZMIZ2 high expression groups, while the infiltration scores of CD4 T cells and DCs were higher (Additional file 1: Fig. S3A). Since these results were based on bioinformatic analysis, we further validated the association between immune cells infiltration and ZMIZ2 expression in HCC through IHC. As the IHC staining shown (Fig. 7C), CD8A, the marker of CD8 T cells was highly expressed in HCC tissues with lower ZMIZ2 expression. In contrast, the markers of CD4 T cells (CD4) and DC cells (CD11c) had the positive correlations with the ZMIZ2 expression. These data were consistent with the bioinformatics results. Furthermore, we found that decreased activated CD8 T cells, enriched CD4 T cells, and enriched DCs in HCC were associated with poor survival outcomes, severing as risk factors for HCC prognosis (Fig. 7B). Notably, patients with high ZMIZ2 expression together with one of these factors had relatively shorter OS times than other subgroups (Additional file 1: Fig. S3B). These results suggest that ZMIZ2 may influence the prognosis of HCC by affecting the infiltration of immune cells.

### ZMIZ2 activated the Wnt/β-catenin signaling pathways through LEF1

**Fig. 8:**
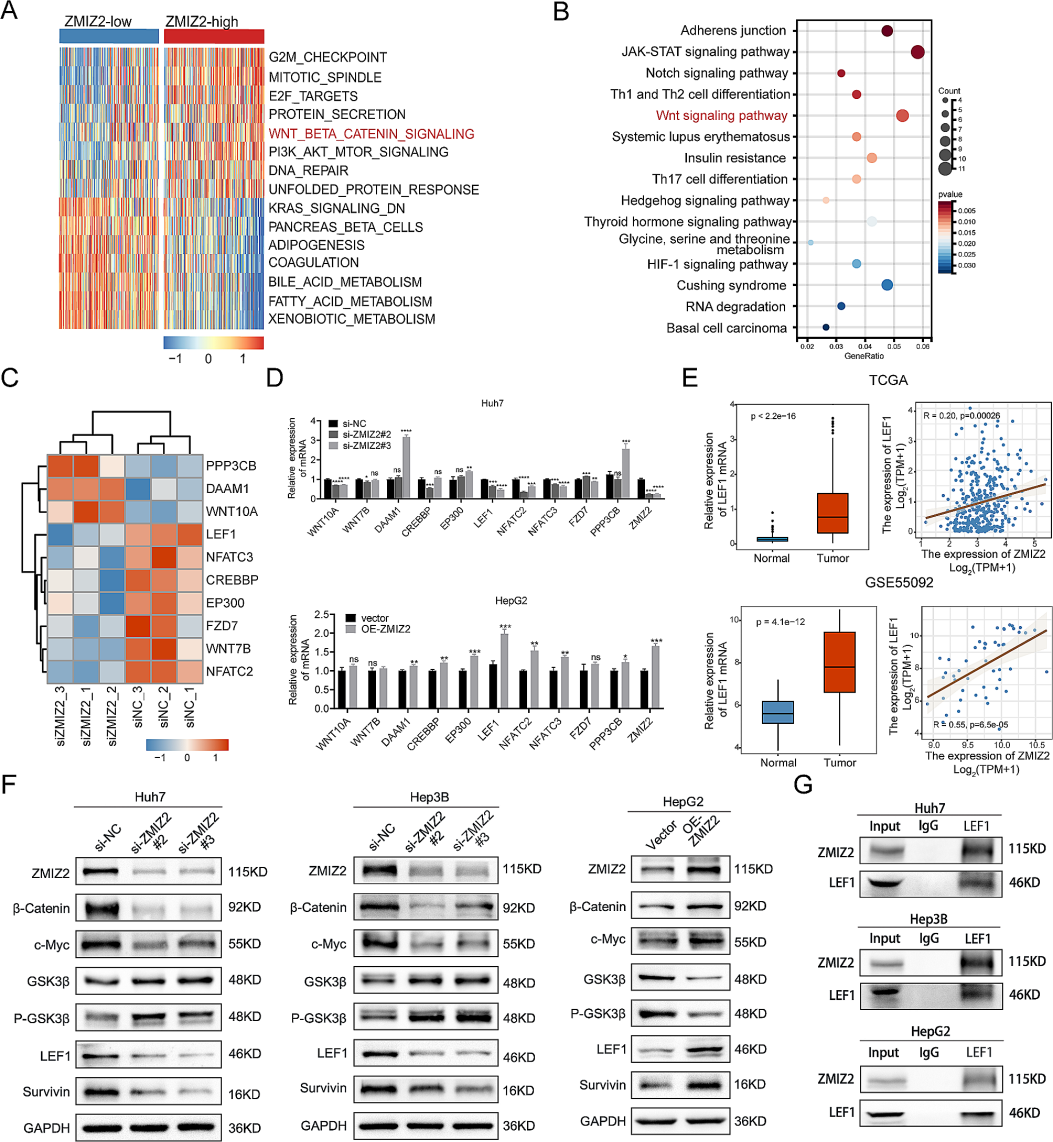
ZMIZ2 activated the Wnt/β-catenin signaling pathways through LEF1. (**A**) Heat map shows the top 15 variated Hallmark pathways between ZMIZ2 expression subgroups by GSVA. (**B**) KEGG analysis for DEGs screened by transcriptome sequencing. (**C**) Heat map shows expressions of the DEGs associated with Wnt/β-catenin signaling pathway between siZMIZ2 and control group. (**D**) The mRNA levels of 10 Wnt/β-catenin pathway related genes with ZMIZ2 knockdown and overexpression were detected by qRT-PCR. (**E**) Expression level of LEF1 in TCGA-LIHC cohort and GSE55092 dataset and correlation of ZMIZ2 expression with LEF1 expression. (**F**) LEF1 and Wnt/β-catenin pathway expression was regulated by ZMIZ2. (**G**) The interaction between ZMIZ2 and LEF1 in HCC cells was investigated using exogenous immunoprecipitation (IP) assay. *p < 0.05; **p < 0.01; ***p < 0.001; **** *p* < 0.0001; ns, not significant

Next, we investigated potential biological distinction between high- and low-ZMIZ2 expression samples. First, differential expression analysis was conducted between high and low ZMIZ2 expression samples separated by the median expression in the TCGA-LIHC cohort (Additional file 1: Fig. S2A). According to GO analysis, the top 200 differential expression genes (DEGs) were significantly enriched in genomic modification-related processes such as chromatin modification, histone modification, and RNA splicing. (Additional file 1: Fig. S2B). Additionally, GSVA analysis revealed an activation of tumor progression-related pathways, including DNA repair, PI3K/AKT/MTOR signaling, and Wnt/β-catenin signaling, in the high-ZMIZ2 group (Fig. 8A). To further investigate the role of ZMIZ2 in HCC, we performed transcriptome sequencing in ZMIZ2-silenced Huh7 cells and control cells, and visualized the DEGs in a heat map (Additional file 1: Fig. S2C). These DEGs (siZMIZ2 versus siNC; adjust. *p* < 0.05) were substantially enriched in the Wnt signaling pathway according to Kyoto Encyclopedia of Genes and Genomes (KEGG) analysis (Fig. 8B), and the siNC samples had higher activation levels of Wnt/β-catenin signaling pathway as confirmed by the GSEA analysis (Additional file 1: Fig. S2D). Taken together, these results suggest that ZMIZ2 plays a crucial role in regulating HCC malignant progression via the Wnt/β-catenin signaling pathway.

Ten DEGs (siZMIZ2 versus siNC) were enriched in the Wnt/β-catenin pathway according to the enrichment analysis, as shown in the heat map (Fig. 8C). We conducted qRT-PCR analysis and observed that three out of the ten genes were the potential targets of ZMIZ2, including LEF1, NFATC2 and NFATC3 (Fig. 8D). Verified by public databases, LEF1 was highly expressed in HCC tissues, which showed a significant positive correlation with the ZMIZ2 expression (Fig. 8E). In contrast, NFATC2 and NFATC3 showed no significant difference between HCC and normal liver tissues (Additional file 1: Fig. S2E). After inhibiting ZMIZ2 expression, the mRNA and protein expression levels of LEF1 were significantly reduced (Fig. 8D, F), and the protein expression levels of positive regulators in the Wnt/β-catenin pathways (Survivin, c-Myc, and β-Catenin) were decreased, whereas the negative regulators (GSK3β and P-GSK3β) were increased (Fig. 8F). An opposite trend was observed in the ZMIZ2-overexpressed HCC cell lines (Fig. 8F). We performed the immunoprecipitation (IP) assay and validated the direct interaction between ZMIZ2 and LEF1 (Fig. 8G). Collectively, these results suggest that ZMIZ2 could activate the Wnt/β-catenin signaling pathways by directly interact with LEF1.

## Discussion

HCC remains a major cause of cancer-related mortality worldwide, despite significant research efforts over decades of years [42, 43]. Therefore, identification of novel prognostic biomarkers and treatment targets of HCC is an urgent need. This study aimed to investigate the biological functions of ZMIZ2 in HCC, which has not been previously described. We found that upregulation of ZMIZ2 was prevalent in HCC and elevated ZMIZ2 expression was associated with worse clinical outcome. Furthermore, our results demonstrate that ZMIZ2 promotes the malignant progression of HCC. These results highlight the potential of ZMIZ2 as a novel prognostic biomarker and therapeutic target for HCC.

ZMIZ2 functions as a transcriptional co-activator that regulates the activity of multiple transcription factors, including p53, Smads and nuclear hormone receptors [14–17]. As a tumor suppressor gene, TP53 is frequently mutant and inactive in liver cancer [44], indicating its inactivation plays a crucial role in the initiation and development of HCC. Smads are downstream targets of the TGF-β signaling pathway, TGF-β1/Smad3 signaling has been demonstrated to promote hepatic gluconeogenesis [45]. ZMIZ2 enhances the transcriptional activity of androgen receptor(AR) [46], which is highly expressed in HCC and is associated with increased tumor recurrence and decreased overall survival [47–50]. These findings suggest that ZMIZ2 plays an important role in HCC development. In this study, we found that ZMIZ2 was highly expressed in HCC tissues and cells, and the expression of ZMIZ2 was a significant independent risk factors for HCC patient survival. In addition, we found that ZMIZ2 promoted the proliferation of primary HCC in vitro and vivo. ZMIZ2 knockdown significantly inhibited the invasion and migration abilities of HCC cells. These results were consistent with previous findings in the breast cancer cells [18] and colorectal cancers [19]. Zou et al. speculated that ZMIZ2 may promote the progression of TNBC by regulating CCL5 to activate the NOD-like receptor signaling pathway and toll-like receptor signaling pathway [18]. Zhu et al. demonstrated that depletion of ZMIZ2 distinctly attenuated colorectal tumorigenesis in mice. ZMIZ2 recruits the enzyme USP7, which deubiquitylates and stabilizes β-catenin, thereby facilitating colorectal tumorigenesis [19]. These data support our conclusion that ZMIZ2 was an oncogene in HCC, and thus it could be a potential prognostic biomarker for this disease.

The tumor suppressor gene TP53 is the molecular hub of multiple crucial signaling pathways, and recent results of whole genome and RNA sequencing analysis for 254 HCC samples also suggested that TP53 was the most frequently mutated gene in HCC [6]. Our research found that HCC patients with high ZMIZ2 expression had higher mutation rate of TP53 and poorer overall survival outcomes, suggesting that ZMIZ2 overexpression and TP53 mutations may jointly affect the development and prognosis of HCC. In HCC, TP53 also shows a high mutation rate and is closely associated with poor prognosis [51, 52], consistent with our analysis. A growing number of studies have indicated a correlation between tumor immune infiltration and cancer progression [53, 54]. Lin et al. found that circRanGAP1 accelerated the HCC progression by regulating the miR-27b-3p/NRAS/ERK axis and affecting the infiltration levels of tumor-associated macrophages [8]. Similarly, we observed a correlation between ZMIZ2 expression and immune infiltration in HCC, which suggested that ZMIZ2 may affect the infiltration of immune cells.

The Wnt/β-catenin signaling pathway regulates the proliferation and differentiation of stem cells and also participates in the expansion of cancer cells. Accumulating evidence has implicated critical roles for both ZMIZ proteins and Wnt/β-catenin signaling pathways in development and tumorigenesis. ZMIZ1 is a coactivator of Notch1 that induces TCF-1 to form a complex with β-catenin, leading to transcriptional activation of target genes via the classical Wnt pathway [55]. In the prostate cancer, ZMIZ2 interacts with β-catenin and improves β-catenin-mediated transcriptions [13]. A similar effect was found in our study. GSEA and transcriptome sequencing analysis showed that ZMIZ2 knockdown had important effects on genes that are mainly related to Wnt/β-catenin signaling pathway. At present, the potential interaction between ZMIZ2 and other members of the Wnt pathway remains unclear. LEF1, as a key component of the Wnt/β-catenin signaling, plays an important role in HCC tumorigenesis, differentiation, and cell survival. Overexpression of LEF1 has been frequently observed in primary liver cancer tissues, and it promotes stemness and poor differentiation of hepatocellular carcinoma by directly activating the NOTCH pathway [26]. LEF1 can increase self-renewal and invasiveness in HCC cells through transcriptional regulation of Oct4 and EMT regulators [22]. Our study suggests that ZMIZ2 interacts with LEF1, therefore activates the Wnt/β-catenin signaling pathway. However, the binding site between ZMIZ2 and LEF1 warrants further studies.

## Conclusion

**Fig. 9:**
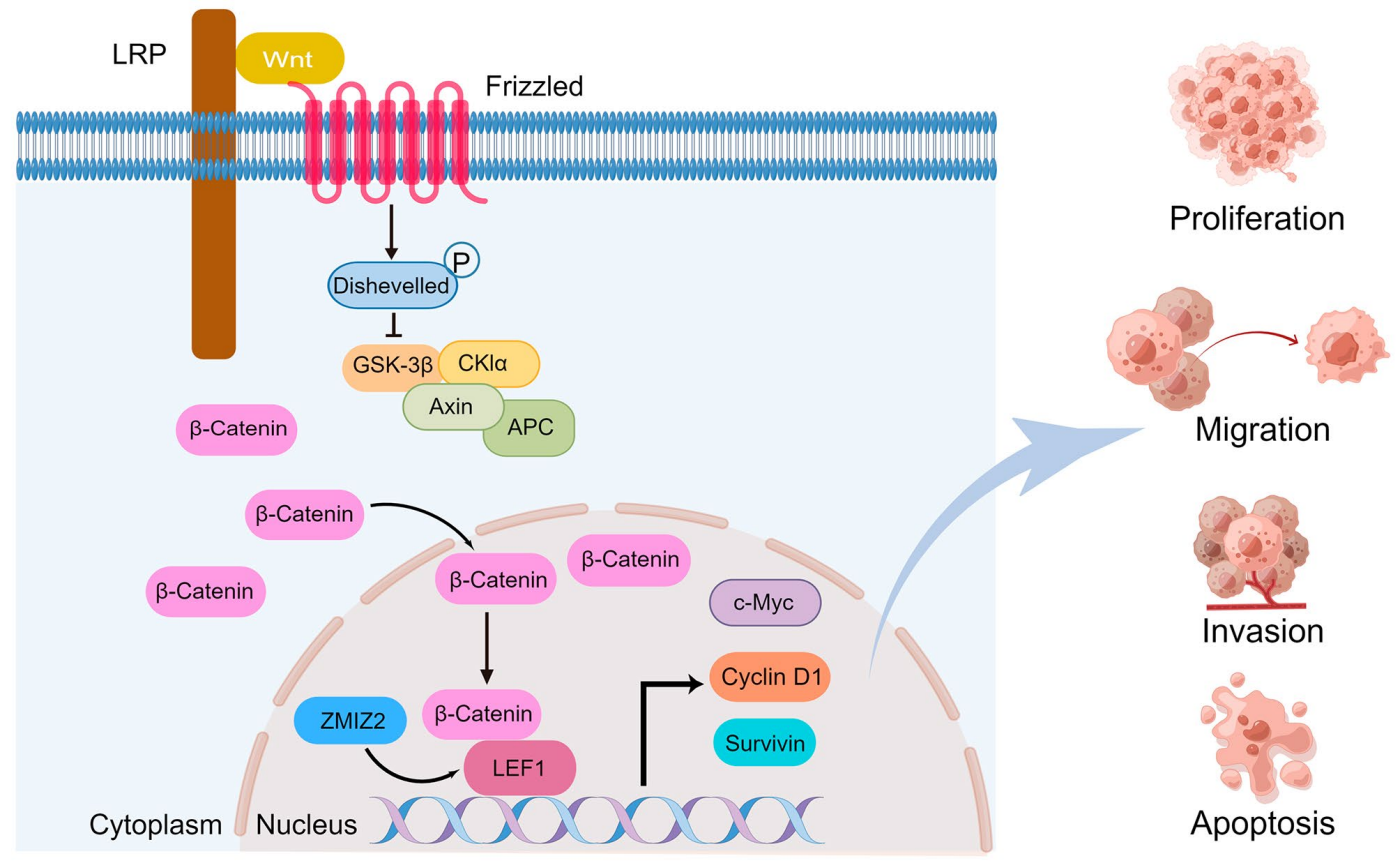
Schematic illustration of the role and molecular mechanisms of ZMIZ2 in HCC. ZMIZ2 promotes HCC cell proliferation, migration, and invasion by activating the Wnt/β-catenin signaling pathway through the upregulation of LEF1

Our study suggests that ZMIZ2 is significantly upregulated in HCC tissues and is associated with poor prognosis in HCC patients. Mechanistically, ZMIZ2 promotes HCC cell proliferation, migration, and invasion by activating the Wnt/β-catenin signaling pathway through the upregulation of LEF1 (Fig. 9). Therefore, ZMIZ2 may serve as a potential prognostic biomarker and therapeutic target for HCC.

### Electronic supplementary material

Below is the link to the electronic supplementary material.

Additional file 1

Additional file 2

## Data Availability

The datasets used and/or analyzed during the current study are available from the corresponding author on reasonable request.

## Abbreviations

AR: Androgen receptor
CCK8: Cell counting kit-8
DEGs: Differential expression genes
DFI: Disease-free interval
GEO: Gene expression omnibus
GO: Gene ontology
GSEA: Gene set enrichment analysis
GSVA: Gene set variation analysis
HCC: Hepatocellular carcinoma
IHC: Immunohistochemistry
LEF1: Lymphoid enhancer-binding factor-1
OS: Overall survival
PFI: Progression-free interval
TAD: Transcription activation domain
TCGA: The cancer genome atlas
TERT: Telomere reverse transcriptase
TMB: Tumor mutation burden
TME: Tumor microenvironment
ZMIZ2: Zinc finger MIZ type containing 2
