# Supplementary material for: ZMIZ2 facilitates hepatocellular carcinoma progression via LEF1 mediated activation of Wnt/β-catenin pathway
Source: Exp Hematol Oncol. 2024 Jan 22;13:5. doi: 10.1186/s40164-024-00475-w (PMC10802047; doi:10.1186/s40164-024-00475-w)
Supplement: Supplementary file 1 — Additional file 1 [file 40164_2024_475_MOESM1_ESM.docx]

**Supplementary Figures**


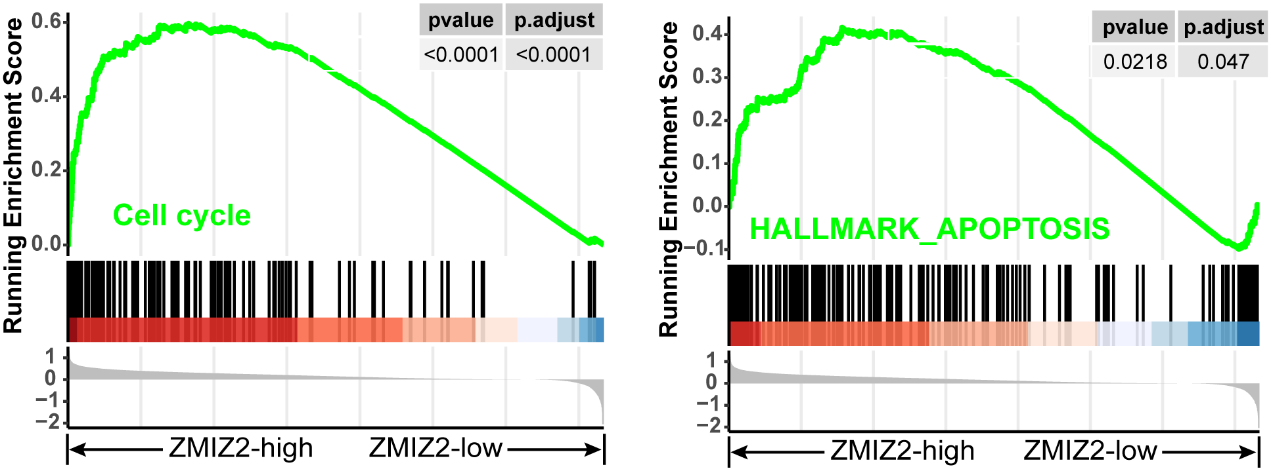


**Figure S1** Data in the TCGA cohort showed that patients with high ZMIZ2 expression were significantly enriched in the cell cycle signaling and apoptosis pathways.

**
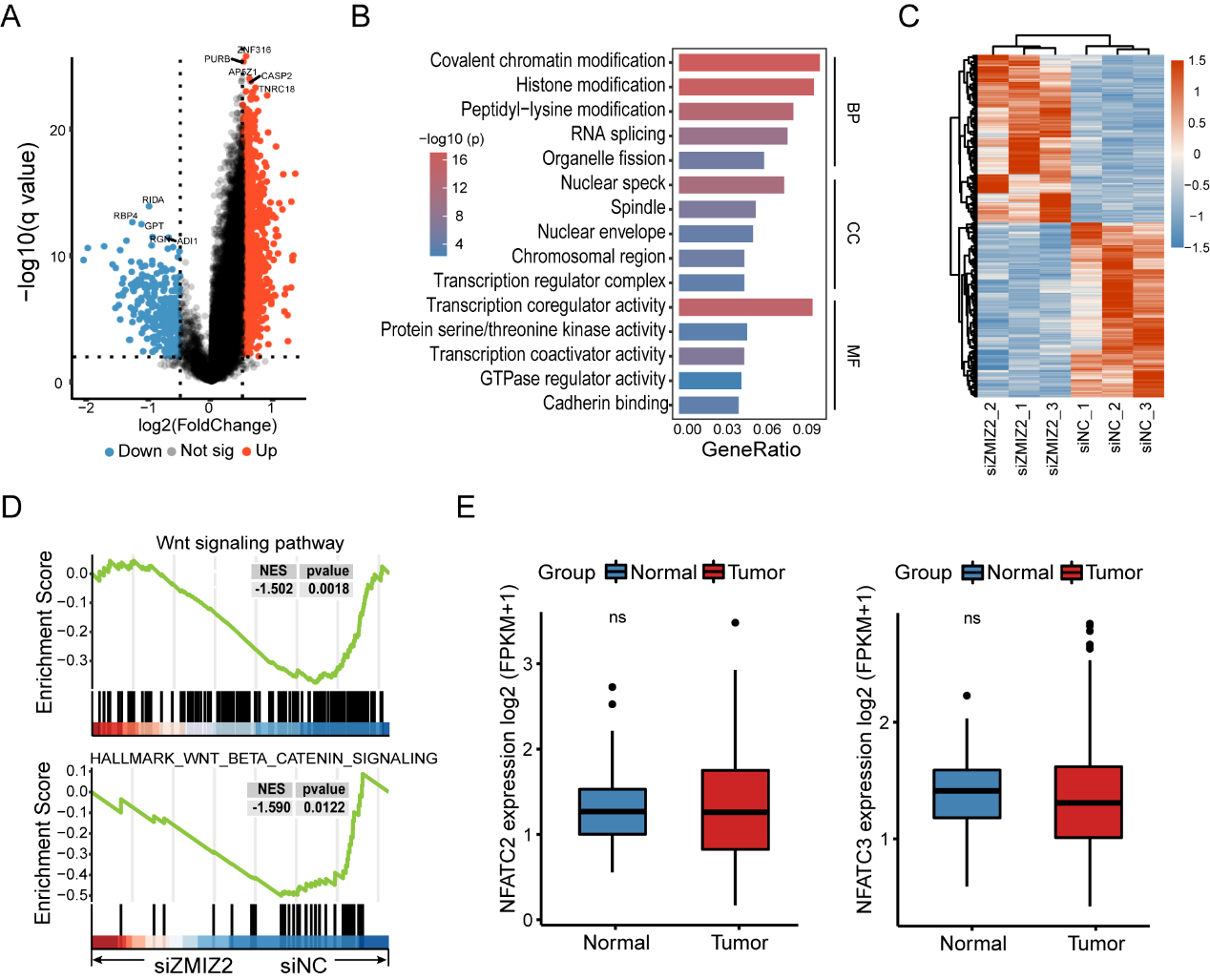
**

**Figure S2** (A) Differential expression analysis for high expression versus low expression groups of ZMIZ2 (separated by median). (B) GO analysis based on the top 200 DEGs. (C) Transcriptome sequencing was performed in Huh7 with silencing ZMIZ2 (siZMIZ2) and the control group, and the results were visualized in a heat map. (D) GSEA plots shows the control group was significant activated in Wnt/β-catenin signaling pathway compared with the siZMIZ2 group. (E)Expression level of NFATC2 and NFATC3 in the TCGA-LIHC cohort.


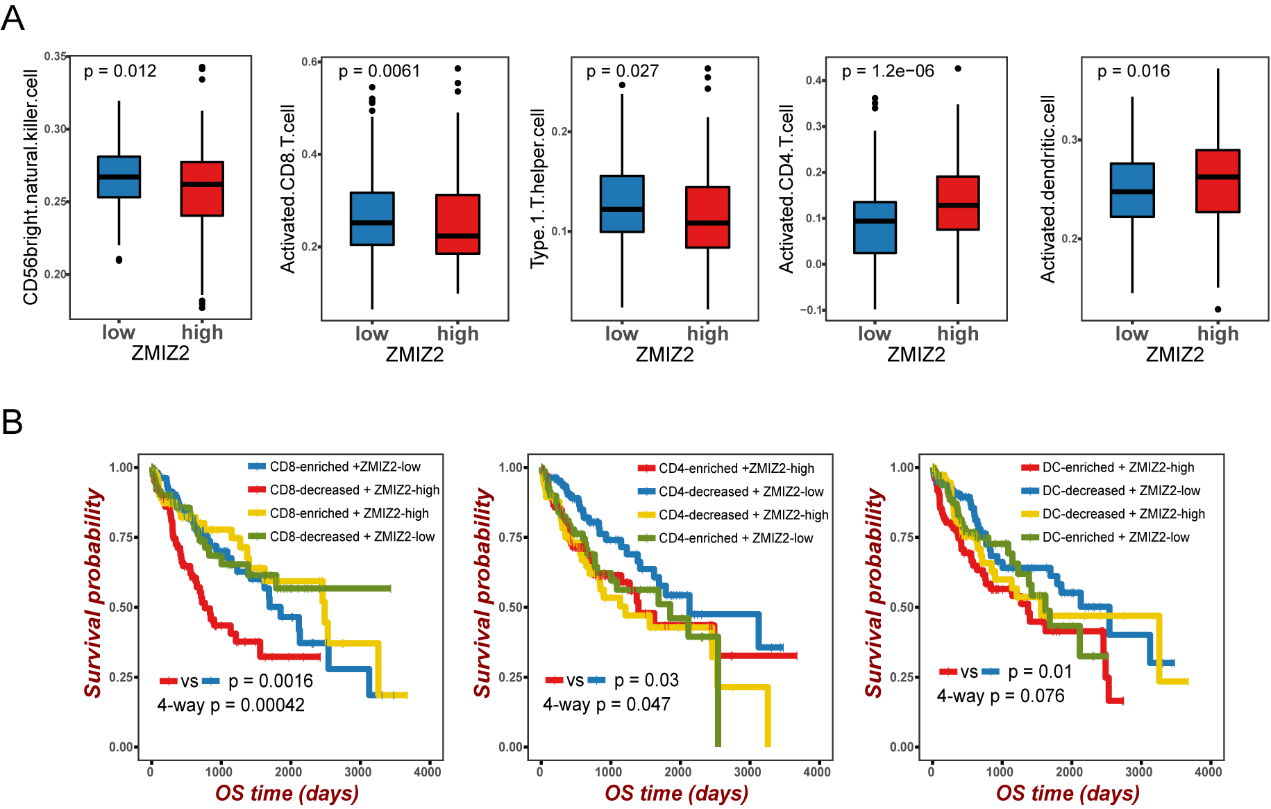


**Figure S3** (A) Box plots showing infiltration levels of several representative immune cells between ZMIZ2 high and low expression groups. (B) Kaplan-Meier curves show the combined effect of several immune cell infiltrations and ZMIZ2 expression on HCC overall survival.
