# Supplementary material for: ZMIZ2 facilitates hepatocellular carcinoma progression via LEF1 mediated activation of Wnt/β-catenin pathway
Source: Exp Hematol Oncol. 2024 Jan 22;13:5. doi: 10.1186/s40164-024-00475-w (PMC10802047; doi:10.1186/s40164-024-00475-w)
Supplement: Supplementary file 2 — Additional file 2 [file 40164_2024_475_MOESM2_ESM.docx]

**Supplementary Table**

**Table S1. List of antibodies used in this study.**

| **Primary Antibody** | **Company** | **Cat.** | **Host** | **Clone** | **Dilution Factor** |
| --- | --- | --- | --- | --- | --- |
| GAPDH | Proteintech | 60004-1-1g | Mouse | Monoclonal | WB 1:1000 |
| ZMIZ2 | Atlas | HPA040716 | Rabbit | Polyclonal | IHC 1:30 |
| ZMIZ2 | ABclonal | A17301 | Rabbit | Polyclonal | WB 1:1000 |
| LEF1 | ABclonal | A0909 | Rabbit | Polyclonal | WB 1:1000  IP 1:50 |
| LEF1 | Proteintech | 14972-1-AP | Rabbit | Polyclonal | IHC 1:200 |
| Bax | CST | 5023 | Rabbit | Monoclonal | WB 1:1000 |
| Bcl-2 | CST | 15071 | Mouse | Monoclonal | WB 1:1000 |
| P21 | Proteintech | 10355-1-AP | Rabbit | Polyclonal | WB 1:1000 |
| P27 | Proteintech | 25614-1-AP | Rabbit | Polyclonal | WB 1:1000 |
| P53 | CST | 2527 | Rabbit | Monoclonal | WB 1:1000 |
| Cyclin D1 | Proteintech | 22337-1-AP | Rabbit | Polyclonal | WB 1:1000 |
| Cyclin B1 | ABclonal | A2056 | Rabbit | Polyclonal | WB 1:1000 |
| Survivin | Abcam | ab76424 | Rabbit | Monoclonal | WB 1:5000 |
| P-GSK3β | ABclonal | AP1088 | Rabbit | Monoclonal | WB 1:1000 |
| GSK3β | CST | 12456 | Rabbit | Monoclonal | WB 1:1000 |
| c-MYC | Proteintech | 10828-1-AP | Rabbit | Polyclonal | WB 1:2000 |
| β-catenin  Ki67 | CST  Proteintech | 8480  28074-1-AP | Rabbit  Rabbit | Monoclonal  Polyclonal | WB 1:1000  IHC 1:1000 |
| CD4 | Abcam | ab133616 | Rabbit | Monoclonal | IHC 1:500 |
| CD8A | ABclonal | A11856 | Rabbit | Polyclonal | IHC 1:100 |
| CD11c | Abcam | ab52632 | Rabbit | Monoclonal | IHC 1:500 |

**Table S2. List of primer, siRNA sequences used in this study.**

**Primers for siRNA**

| siRNA sequence | sense（5'-3'） | antisense（5'-3'） |
| --- | --- | --- |
| Scramble | UUCUCCGAACGUGUCACGUTT | ACGUGACACGUUCGGAGAATT |
| ZMIZ2-si1 | CCACGGGACUGCAUUAUAATT | UUAUAAUGCAGUCCCGUGGTT |
| ZMIZ2-si2 | GUCCCUUACAUGUCACCAATT | UUGGUGACAUGUAAGGGACTT |
| ZMIZ2-si3 | GCCACAUACAGUGCUUUGATT | UCAAAGCACUGUAUGUGGCTT |

**Quantitative PCR primers**

| Primer sequence | Forward | Reverse |
| --- | --- | --- |
| ZMIZ2 | ATGAACCCTACTGGCATAGGAG | GCCCAGGATACCCATGTTGG |
| GAPDH | CCCCAGCAAGAGCACAAGAG | GCACAGGGTACTTTATTGATGGTAC |
| LEF1 | TGCCAAATATGAATAACGACCCA | GAGAAAAGTGCTCGTCACTGT |
| DAAM1 | AAATTGAAACGGAATCGCAAAC | GCAAGGCAGTGTAATGAAACG |
| NFATC2 | TGCATCTAACCCCATCGAGTG | TGAGGATCATTTGCTGGC |
| NFATC3 | TTCGCACATCTTCATTACCTCC | CCTCGGCTACCTTCAGTTTCAT |
| WNT10A | GGTCAGCACCCAATGACATTC | TGGATGGCGATCTGGATGC |
| WNT7B | CACCTTCCTGCGCATCAAAC | GTCCTCCTCGCAGTAGTTGG |
| EP300  CREBBP | TGCAGGCATGGTTCCAGTTT  CAACCCCAAAAGAGCCAAACT | AGGTAGAGGGCCATTAGAAGTCA  CCTCGTAGAAGCTCCGACAGT |
| PPP3CB | CCCCAACACATCGCTTGACAT | GGCAGCACCCTCATTGATAATTC |
| FZD7 | CAACGGCCTGATGTACTTTAAGG | CATGTCCACCAGGTAGGTGAGA |
